# Association between Fibrinogen-to-Albumin Ratio and Prognosis of Hospitalized Patients with COVID-19: A Systematic Review and Meta-Analysis

**DOI:** 10.3390/diagnostics12071678

**Published:** 2022-07-10

**Authors:** Kuo-Chuan Hung, Yen-Ta Huang, Ying-Jen Chang, Chia-Hung Yu, Li-Kai Wang, Chung-Yi Wu, Ping-Hsin Liu, Sheng-Fu Chiu, Cheuk-Kwan Sun

**Affiliations:** 1 Department of Anesthesiology, Chi Mei Medical Center, Tainan City 71004, Taiwan; ed102605@gmail.com (K.-C.H.); 0201day@yahoo.com.tw (Y.-J.C.); dkntstar@hotmail.com (C.-H.Y.); anesth@gmail.com (L.-K.W.); wcy34300-ad@yahoo.com.hk (C.-Y.W.); 2 Department of Hospital and Health Care Administration, College of Recreation and Health Management, Chia Nan University of Pharmacy and Science, Tainan City 71710, Taiwan; 3 Department of Surgery, National Cheng Kung University Hospital, College of Medicine, National Cheng Kung University, Tainan City 70101, Taiwan; uncleda.huang@gmail.com; 4 Department of Recreation and Health-Care Management, College of Recreation and Health Management, Chia Nan University of Pharmacy and Science, Tainan City 71710, Taiwan; 5 Department of Anesthesiology, E-Da Hospital, Kaohsiung City 82445, Taiwan; neoplasmboy@yahoo.com.tw; 6 Department of Oral and Maxillofacial Surgery, Chi Mei Medical Center, Liouying, Tainan City 73657, Taiwan; 7 Department of Emergency Medicine, E-Da Hospital, Kaohsiung City 82445, Taiwan; 8 College of Medicine, I-Shou University, Kaohsiung City 84001, Taiwan

**Keywords:** fibrinogen-to-albumin ratio, coronavirus disease 2019, mortality, disease severity, meta-analysis, prognosis

## Abstract

Although the fibrinogen-to-albumin ratio (F/R ratio) has been used as an inflammation marker to predict clinical outcomes in patients with cardiovascular diseases, its association with the prognosis of patients with coronavirus disease 2019 (COVID-19) remains unclear. Electronic databases including EMBASE, MEDLINE, Google Scholar, and Cochrane Library were searched from inception to 20 June 2022. The associations of F/R ratio with poor prognosis (defined as the occurrence of mortality or severe disease) were investigated in patients with COVID-19. A total of 10 studies (seven from Turkey, two from China, one from Croatia) involving 3675 patients published between 2020 and 2022 were eligible for quantitative syntheses. Merged results revealed a higher F/R ratio in the poor prognosis group (standardized mean difference: 0.529, *p* < 0.001, I^2^ = 84.8%, eight studies) than that in the good prognosis group. In addition, a high F/R ratio was associated with an increased risk of poor prognosis (odds ratio: 2.684, I^2^ = 59.5%, five studies). Pooled analysis showed a sensitivity of 0.75, specificity of 0.66, and area under curve of 0.77 for poor prognosis prediction. In conclusion, this meta-analysis revealed a positive correlation between F/A ratio and poor prognostic outcomes of COVID-19. Because of the limited number of studies included, further investigations are warranted to support our findings.

## 1. Introduction

The global outbreak of the coronavirus disease 2019 (COVID-19) pandemic has already claimed millions of lives since the end of 2019 [1], and the figure is still on the rise. The clinical manifestations of SARS-CoV-2 infection vary widely, from no apparent symptoms to life-threatening acute respiratory distress syndrome and multiorgan dysfunction [2,3,4]. Of those contracting the disease, 20% may rapidly progress to severe illnesses [2,3,4] and approximately 6% may die [5]. Therefore, previous studies have focused on the identification of sensitive prognostic markers to guide medical resource allocation and ensure timely intervention for those at risk of severe disease [6].

A large-scale meta-analysis enrolling more than 13,000 participants has shown that the serum level of cytokines (i.e., IL-6) could be a potential indicator of disease severity as well as mortality for those diagnosed with COVID-19 [7]. The results of the study are compatible with the reported inflammatory reactions of COVID-19 resulting from unsuppressed immune responses [8,9]. Besides triggering the cytokine pathways, another aspect of the disease is the activation of the coagulation system [10,11], as reflected by elevations in circulating D-dimer and fibrinogen concentrations, but with thrombocytopenia in those with severe disease [12,13]. The serum level of fibrinogen, which is a procoagulant protein synthesized in the liver, is known to increase in inflammatory conditions [14]. Through the action of thrombin (FIIa), fibrinogen is converted to fibrin, which participates in the formation of cross-linked fibrin clots in a coagulation process mediated by FXIIIa. On the other hand, plasmin, which is the active form of plasminogen following its cleavage by plasminogen activators, modulates the process of fibrinolysis, which involves the degradation of fibrin into fibrin degradation products [15]. In addition, previous studies have demonstrated a positive association between circulating fibrinogen levels and mortality from COVID-19 [16,17]. Another readily measurable serum parameter reported to be related to the disease severity of COVID-19 is albumin [18], which is the predominant serum protein that usually serves as an indicator of an individual’s nutritional status. Moreover, the circulating album level has been shown to decrease with progressive inflammation [19].

Taking into account the roles of inflammation and nutrition in a patient’s prognosis, the fibrinogen-to-albumin ratio (F/A ratio) was introduced as a prognostic factor for patients with cardiovascular diseases and cancers, with an increase indicating a microinflammatory condition [20,21]. Given that immunity plays a critical role in COVID-19 progression [22,23], a number of studies have addressed the accuracy of the F/A ratio in predicting the severity and mortality of COVID-19 [12,24,25]. Nevertheless, evidence in support of the prognostic value of the F/A ratio was obtained from previous single-center observational studies. Therefore, through systematically reviewing the pooled evidence, the present meta-analysis aimed at examining the association of the F/A ratio with prognostic outcomes among patients contracting COVID-19. The results could provide practical clinical guidance for clinicians, not only to enable the timely delivery of medical care but also to aid in effective resource allocation, which are crucial to the treatment of patients diagnosed with COVID-19 [6].

## 2. Materials and Methods

This systematic review is reported according to the Preferred Reporting Items for Systematic Reviews and Meta-Analysis statement. Our study protocol was registered in the International Prospective Register of Systematic Reviews (CRD42022340682). Two authors independently conducted study selection, data collection, and risk of bias evaluation to improve the quality of the current meta-analysis. All disagreements were resolved through discussion. The protocol and procedures of the present investigation have been detailed in our previous report [26,27].

### 2.1. Data Sources and Literature Search

With relevant MeSH terms and keywords, we searched for observational studies investigating the association of the F/A ratio with prognostic outcomes among adults diagnosed with COVID-19 on 20 June 2022, from the following databases: Embase, Medline, Google Scholar, and Cochrane Library. Taking Medline as an example, Supplemental Table S1 shows the procedure of the literature search. We placed no restrictions on language, sample size, year of publication, and country during the database search. To ensure the completeness of our search, the lists of references of the retrieved articles and published meta-analyses were examined to identify potentially eligible studies.

### 2.2. Study Sreening

Observational studies fulfilling the following inclusion criteria were considered eligible: (1) cohort studies, case-controlled studies, and those adopting a cross-sectional design; (2) hospitalized adults with confirmed diagnosis of COVID-19; (3) available baseline F/A ratio at hospital admission; (4) available prognostic outcomes including events of mortality and disease severity; and (5) provision of adequate details necessary for calculating or extracting odds ratios (ORs) and 95% confidence intervals (CIs). Studies (1) containing duplicated data; (2) focusing on children or patients not hospitalized; (3) being published in the form of symposium abstracts, case reports, systematic reviews, editorials or commentaries, animal experiments, and other forms of publication instead of original clinical works were excluded.

### 2.3. Data Collection

Information about the first author, year of publication, age of participants, gender prevalence, number of patients, indicators of prognosis, such as mortality and disease severity, F/A ratio, and country was collected from each study. Moreover, the OR and 95% CI were obtained from matched or adjusted data. We used the adjusted ORs for studies with information on both unadjusted and adjusted ORs. The calculation of ORs for categorical variable generated from a continuous parameter through dichotomization was based on the number of individuals in the study and control groups with prognostic factor exposure (e.g., F/A ratio), in accordance with the cut-off value described in the article. We contacted the authors of the included studies with missing data in an attempt to retrieve additional information.

### 2.4. Definitions and Outcomes

The primary outcome was the mean difference in F/A ratio in poor and good prognosis groups, while the secondary outcomes included the risk of poor prognosis in patients with a high F/A ratio, as well as the diagnostic efficacy of the F/A ratio for predicting poor prognostic outcomes. Patients with poor prognostic outcomes (i.e., poor prognosis group) were defined as those who had mortality or severe disease, while the good prognosis group referred to those who did not have poor prognostic outcomes. The definition of disease severity was according to that defined in each study. The correlations of other risk factors (e.g., hypertension) with prognostic outcomes were not investigated in the current meta-analysis due to the limited number of available studies.

### 2.5. Risk of Bias Assessment for the Included Studies

Two authors independently evaluated the risk of bias in each study based on the six domains described in the Quality in Prognostic Studies (QUIPS) tool, including study participation, outcome quantification, study attrition, prognostic factor measurement, adjustment for other prognostic factors, as well as statistical analysis and reporting [28]. For each study, the risk for each domain was judged as low, unclear, or high. We considered the overall risk of bias to be low if all (or most) of the domains in the study were deemed low or low to moderate [29].

### 2.6. Data Synthesis and Analysis

Due to the inclusion of observational studies in the present meta-analysis, an overall OR generated with a random-effects model was adopted as the major summary measure of effect size. Effect size heterogeneity was evaluated using I^2^ statistics, with significant heterogeneity being defined as a value of I^2^ above 50% [30]. Sensitivity analysis was conducted by deleting a study each time to test the robustness and reliability of our acquired evidence. Funnel plots and Egger’s tests were used to detect potential publication bias. All statistical analyses were performed using the comprehensive Meta-Analysis (CMA) V3 software (Biostat, Englewood, NJ, USA).

To evaluate the accuracy of the F/A ratio in the prediction of poor prognosis, we computed pooled estimates of sensitivity and specificity using the bivariate model [31]. The area under the curve (AUC) from a hierarchical summary receiver operating characteristic (hsROC) curve was used to assess the overall accuracy. The MIDAS command in Stata 15 (StataCorp LLC., College Station, TX, USA) was used to generate forest plots on pooled sensitivity/specificity and sROC curves, as well as Deeks’ funnel plot for evaluating publication bias. Statistical significance was set at a probability value less than 0.05.

## 3. Results

### 3.1. Study Selection

**Figure 1:**
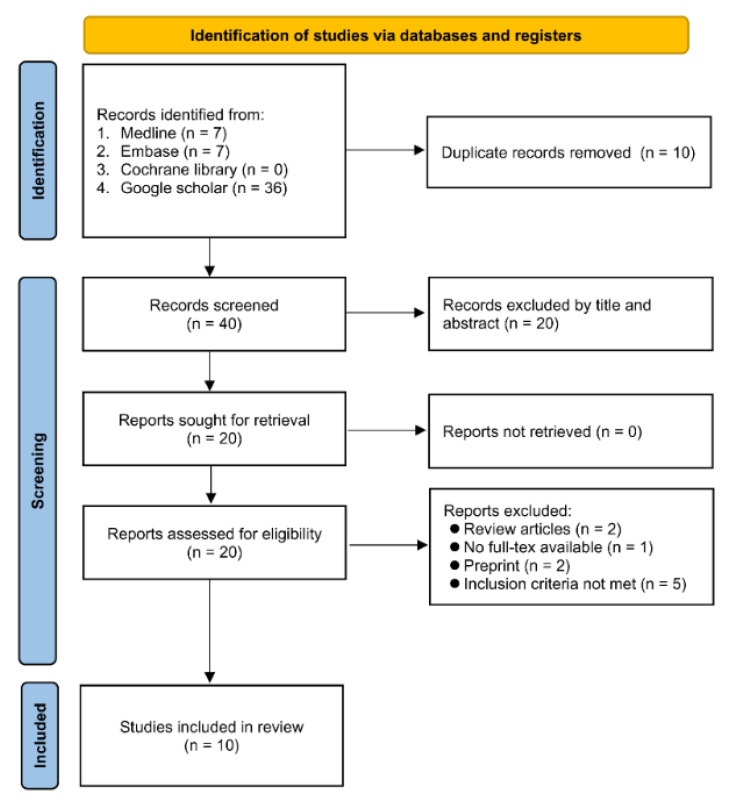
Flow chart for inclusion and exclusion.

A flow diagram summarizing the study selection process is shown in Figure 1. Of a total of 50 records acquired through the initial database search, 10 and 20 were excluded because of data duplication and ineligibility based on their titles or abstracts, respectively. Of the 20 remaining articles, 10 were excluded following full-text review for different reasons (Figure 1). Finally, 10 studies recruiting a total of 3675 patients published between 2020 and 2022 were deemed eligible for quantitative syntheses [12,24,25,32,33,34,35,36,37,38].

### 3.2. Characteristics of Studies and Risk of Bias

The characteristics of the included studies are summarized in Table 1. The included studies were conducted mainly in Turkey (seven studies) [24,25,32,33,34,35,37], while the other three studies were conducted in China (two studies) [12,38] and Croatia (one study) [36]. Five studies recruited hospitalized patients, without providing details about the units to which they were admitted [12,32,35,37,38], while three studies focused on those admitted to the intensive care unit (ICU) [24,33,36]. Of the other two studies, one investigated assorted hospitalized and ICU patients [25] and one researched those admitted through the emergency department [34]. There were over 100 participants in each study, with the number ranging between 113 and 717. The age of the participants ranged between 44 and 76 years, with a male prevalence of 44–65%. Details regarding gender distribution were not mentioned in one study [36]. Six studies investigated the association between the F/A ratio and mortality in patients with COVID-19 [24,25,33,34,35,36], while the other four focused on the relationship between the F/A ratio and disease severity [12,32,37,38]. Three studies defined disease severity as patients with respiratory distress or organ dysfunction [12,32,37], while one study defined disease severity as those with respiratory distress [38]. Seven studies provided the cut-off value for the F/A ratio to predict prognostic outcomes (i.e., 9–14) [12,25,32,34,35,37,38], while relevant information was not available in the other three studies [24,33,36].

**Figure 2:**
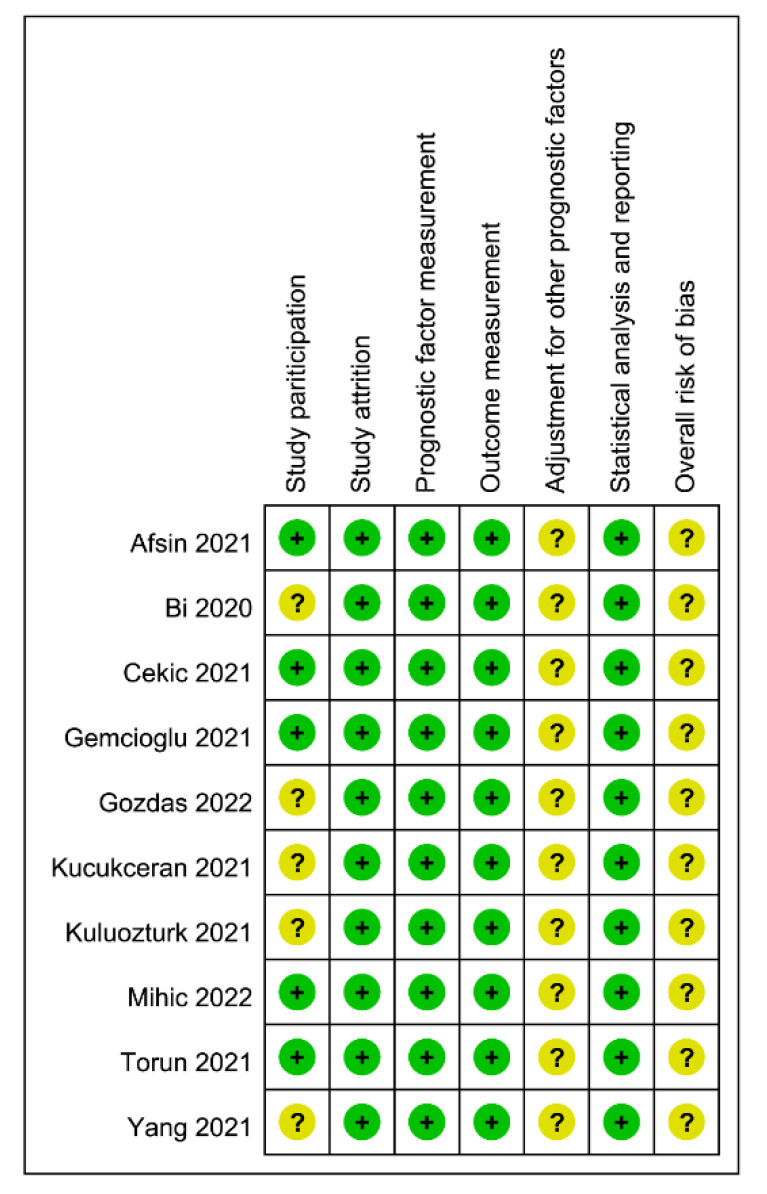
Risks of bias based on assessment with the Quality in Prognostic Studies (QUIPS) tool [12,24,25,32,33,34,35,36,37,38].

The risks of bias based on the QUIPS tool are shown in Figure 2. For five studies, the risk of bias of study participation was deemed unclear due to the significant difference in patient age between the two groups [12,33,34,35,38]. Due to the adoption of a retrospective study design in all of the included studies, the adjustment for other prognostic factors may not be adequate; therefore, the risk of bias for this domain was considered to be unclear in all studies. The overall risk of bias was deemed unclear in all studies.

### 3.3. Data Analysis

#### 3.3.1. Association of Fibrinogen-to-Albumin Ratio with Prognosis

**Figure 3:**
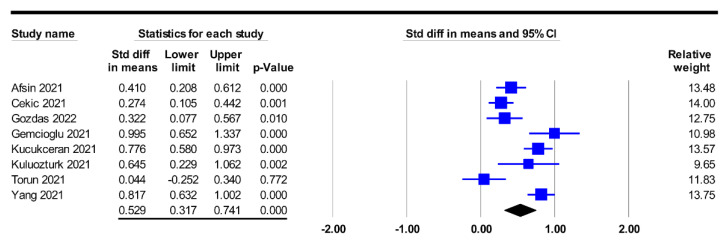
Forest plot comparing fibrinogen-to-albumin (F/A) ratio between poor and good prognosis groups, showing a higher F/A ratio in the former compared to the latter (SMD: 0.529, 95% CI: 0.317 to 0.741, *p* < 0.001, I^2^ = 84.8%) [24,25,32,33,34,35,37,38]. Std diff, standardized difference; CI, confidence interval.

**Figure 4:**
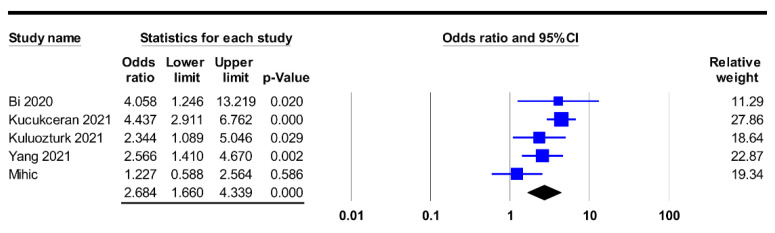
Forest plot demonstrating a positive correlation between risk of poor prognosis and fibrinogen-to-albumin ratio as a binary parameter (odds ratio: 2.684, 95% CI: 1.66 to 4.339, *p* < 0.001, I^2^ = 59.5%) [12,34,35,36,38]. CI, confidence interval.

**Figure 5:**
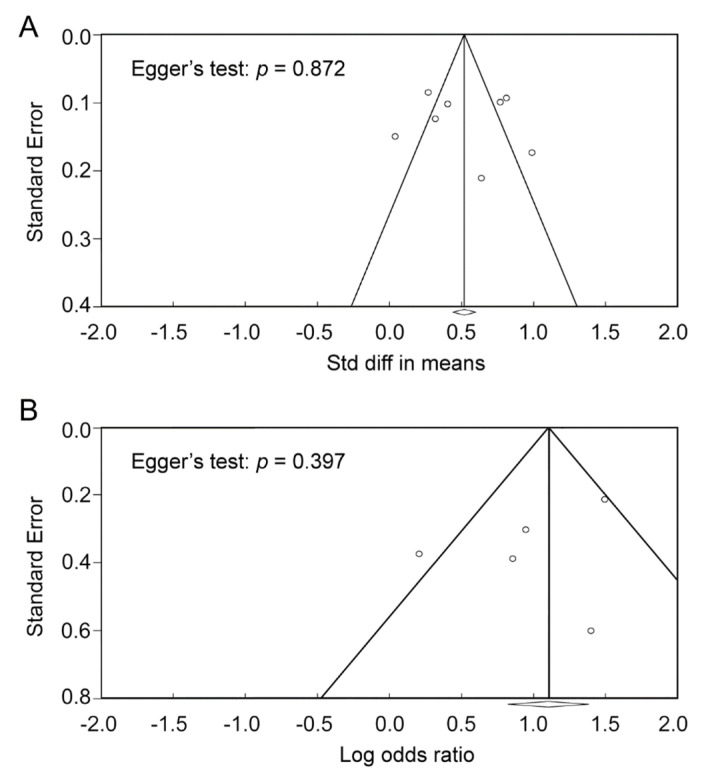
Funnel plot with Egger’s test showing no publication bias in (**A**) the mean difference in fibrinogen-to-albumin ratio (F/A ratio) in both groups [24,25,32,33,34,35,37,38], and (**B**) risk of poor prognosis in patients with high F/A ratio [12,34,35,36,38].

Based on the F/A ratio in the poor prognosis and good prognosis groups, the merged results revealed a higher F/A ratio among patients in the poor prognosis group (SMD: 0.529, 95% CI: 0.317 to 0.741, *p* < 0.001, I^2^ = 84.8%, eight studies) compared to that in the good prognosis group (Figure 3) [24,25,32,33,34,35,37,38]. An investigation into the correlation between the F/A ratio as binary variable (i.e., low vs. high) and the risk of poor prognosis in five articles with available information also showed an association of a higher F/A ratio with a higher risk of poor prognosis (OR: 2.684, 95% CI: 1.66 to 4.339, *p* < 0.001, I^2^ = 59.5%) (Figure 4) [12,34,35,36,38]. Sensitivity analysis demonstrated the robustness of these two outcomes, without significant publication bias (Figure 5).

#### 3.3.2. The Use of Fibrinogen-to-Albumin Ratio for Predicting Poor Prognosis: Pooled Sensitivity/Specificity Estimates and sROC

**Figure 6:**
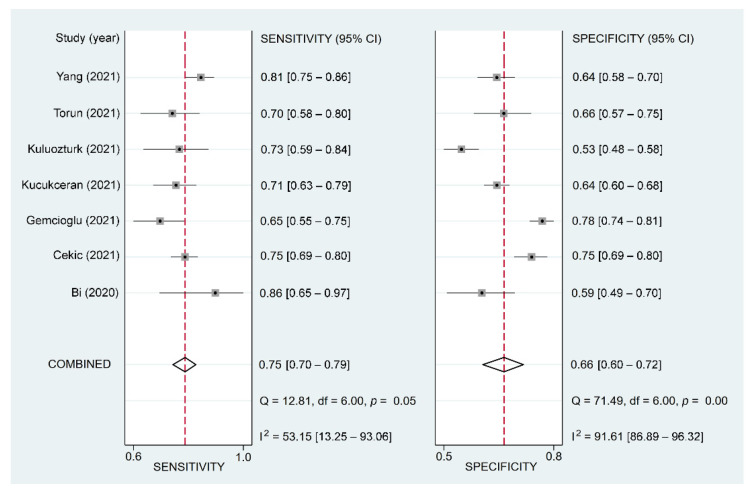
Forest plots comparing the sensitivity and specificity of using fibrinogen-to-albumin ratio for predicting poor prognosis in patients with COVID-19 among the included studies [12,25,32,34,35,37,38].

**Figure 7:**
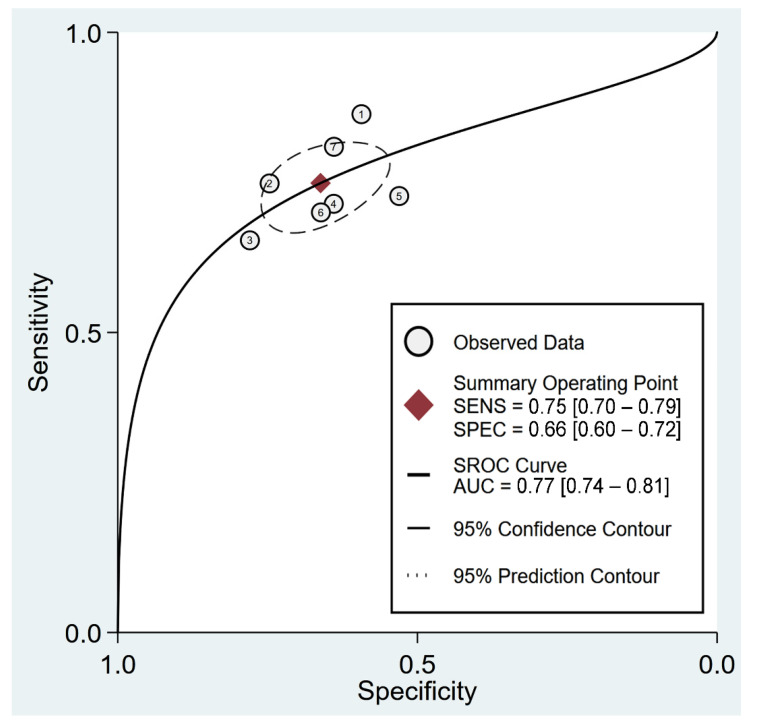
Hierarchical summary receiver operating characteristic (hsROC) curves of using fibrinogen-to-albumin (F/A) ratio for predicting in-hospital mortality among patients diagnosed with COVID-19 [12,25,32,34,35,37,38]. SENS, sensitivity; SPEC, specificity; SROC, summary receiver operating characteristic; AUC, area under the curve.

Using the F/A ratio for the prediction of poor prognosis gave a pooled sensitivity and specificity of 0.75 (95% CI = 0.7–0.79; I2 = 53.15%) and 0.66 (95% CI = 0.6–0.72; I^2^ = 91.61%), respectively (Figure 6) [12,25,32,34,35,37,38]. For each study, linear regression for sROC following mathematical adjustment of true and false positivity (i.e., 1-specificity) showed an AUC of 0.77 (95% CI: 0.74–0.81) (Figure 7). Deeks’ funnel plot asymmetry test did not demonstrate notable publication bias (*p* = 0.41).

## 4. Discussion

The ever-increasing number of patients diagnosed with COVID-19 has highlighted the importance of identifying those with high risks of mortality and complications to enable the rational allocation of healthcare resources and to implement timely individualized therapeutic strategies [39]. Our results not only revealed a higher F/A ratio (SMD: 0.529) in patients with poor prognosis but also showed a nearly three-fold increase in the risk of poor prognosis among patients diagnosed with COVID-19. Considering the simplicity of computation, our results suggested that the F/A ratio may be an indicator for cost-effective medical resource allocation when the global healthcare supplies are overwhelmed by the pandemic.

A link between coagulation–fibrinolytic cascades and inflammatory response has been reported, with fibrinolysis being identified as an indicator of the onset of systemic inflammation [40]. In addition to the well-known roles of fibrinogen in promoting platelet aggregation, increasing plasma viscosity, and causing erythrocyte aggregation [41], previous studies have shown that it is a reactive protein that indicates the acute phase of systemic inflammation, including that arising from injury and infection [14,42]. Fibrinogen acts as a scaffold for platelet aggregation through the activated form of integrin αIIbβ3 (also known as glycoprotein IIb/IIIa). Platelet aggregation via fibrinogen cross-linking offers an initial hemostatic barrier following blood vessel injury as part of the rapid primary hemostatic response. The main role of fibrinogen in hemostasis is to reinforce the platelet plug after conversion into an insoluble fibrin polymer by thrombin cleavage of fibrinopeptides A and B. The fibrin polymer traps red blood cells and platelets, forming a stable fibrin plug that stops bleeding from the site of injury [43,44]. Indeed, fibrinogen has been found to trigger the production of pro-inflammatory cytokines, thereby contributing to the development of inflammation [14]. A previous meta-analysis has identified an increased level of fibrinogen as a risk factor for poor prognosis in patients diagnosed with COVID-19 [45]. Albumin, which is synthesized and secreted by hepatocytes, not only is the major component responsible for maintaining intravascular colloidal pressure but also serves as a transporter for many insoluble small molecules of organic and inorganic substances and a nutritional indicator. Previous studies also revealed its role in the inflammation process [46], during which endothelial barrier dysfunction results in albumin hyperpermeability and tissue edema [47]. Consistently, a recent meta-analysis identified a low circulating serum albumin level as an independent predictor of poor prognosis in patients with COVID-19 [48]. Evidence in support of the association of fibrinogen and albumin with prognosis in patients with COVID-19 [45,47] has prompted an exploration of the prognostic value of the F/A ratio in this clinical setting [24,35,38]. In the current meta-analysis, our results supported a role of this inflammation marker in predicting a poor prognosis in patients with COVID-19.

Although previous observational studies have reported several other inflammatory or coagulation-related makers for the prediction of prognostic outcomes in patients contracting COVID-19 [49,50], our meta-analysis is the first to investigate the association of the F/A ratio with poor prognosis. A previous meta-analysis involving more than two thousand patients revealed significantly higher CRP levels among those who succumbed to COVID-19 than in survivors [51]. In addition, another even larger meta-analysis on over 13,000 participants recruited in 77 observational studies has demonstrated a role of the circulating level of cytokines (i.e., IL-6) in predicting severity and mortality in those diagnosed with COVID-19 [7], further underscoring a detrimental prognostic role of inflammation. In respect to the coagulation–inflammation cascade, previous observational studies have identified D-dimer and fibrinogen as poor prognostic indicators in those contracting COVID-19 [25,49,52]. Among these parameters, receiver operating characteristic (ROC) curve analysis in a previous observational study showed the superior accuracy of the F/A ratio (area under curve (AUC) = 0.808) in predicting disease severity and mortality compared with other parameters (i.e., C-reactive protein: AUC = 0.735; fibrinogen: AUC = 0.606; albumin: AUC = 0.257) [25]. The findings of the mentioned study implied the clinical value of the F/A ratio in providing prognostic information to guide decision-making among patients with severe COVID-19 infection.

The relatively high heterogeneities in both the mean difference and ORs regarding the correlation between the F/A ratio and poor prognosis in our meta-analysis (I^2^ = 84.8% and 59.5%, respectively) (Figure 3 and Figure 4) may be attributed to variations in study population (e.g., ICU patients or hospitalized patients), hospital setting, the age and gender distribution of the participants, definition of disease severity, F/A ratio cut-off value, and treatment protocol across the included studies. Accordingly, we used a random-effects model to minimize the potential bias. Despite the high heterogeneities, sensitivity analysis demonstrated the robustness of our results. Furthermore, the lack of significant publication bias in our results further supports the association between a high F/A ratio and poor prognostic outcomes in patients diagnosed with COVID-19.

There were two advantages in the current meta-analysis. First, the inclusion of different study populations (e.g., ICU vs. assorted hospitalized patients) in the current meta-analysis suggests the wide application of our findings. Second, there is a known ethnic impact on the prognosis of COVID-19, with lower mortality and disease severity being reported in Asians than in other populations [53,54]. The high homogeneity from the inclusion of the majority of studies from a single country (e.g., Turkey) may minimize the ethnic biases in the present meta-analysis.

The current study had several limitations. First, the retrospective nature of our included studies increased the susceptibility of our results to a number of confounding factors. For example, the lack of information about known determinants of disease severity such as viral load and treatment strategies may introduce bias to our findings [55]. Second, given the known fluctuation in the F/A ratio after hospital admission (e.g., highest at 6–10 days after admission) [12], the use of data on hospital admission in the current study may be a source of bias. Third, given the significant variations in disease severity and mortality with geographical locations and different phases of the pandemic [5], the inclusion of predominantly Turkish studies may not justify the extrapolation of our results to other countries and ethnic groups. Fourth, although comorbidities have been found to contribute to mortality and severity in patients diagnosed with COVID-19 [56,57], the lack of relevant information precluded a subgroup analysis of their potential impacts on our study outcomes. Fifth, of the 10 included studies, only four provided relevant information about the equipment and procedures for fibrinogen measurement [24,33,35,37]. Therefore, potential bias arising from different methods for fibrinogen quantification could not be ruled out. Sixth, although four of our 10 included studies focused on patients admitted to the ICU, none of them provided information about coexisting infections (e.g., ventilator-associated pneumonia, catheter-related bloodstream infections). Therefore, potential impacts of such infections on our study outcomes remain unclear. Finally, the reliability of our findings may be blemished by variations in the cut-off value of the F/A ratio for prognostic prediction, as well as the definition of disease severity across the included studies.

## 5. Conclusions

This meta-analysis of 10 observational studies on 3675 hospitalized patients diagnosed with COVID-19 showed a positive association of the fibrinogen-to-albumin ratio with poor prognostic outcomes. As global healthcare is already overwhelmed by the pandemic, a simple, cost-effective predictor may be crucial to the identification of patients at risk of prognostic outcomes, to enable the effective allocation of medical resources. Further large-scale studies incorporating this readily obtainable parameter into clinical practice are required to support its prognostic value in patients contracting COVID-19.

## Figures and Tables

**Table 1:** Characteristics of studies (*n* = 10).

Author (Year)	Population	Age (Years)	Male (%)	*n*	Outcome	Cut-Off Value for F/A Ratio	Country
Afsin (2021)	ICU patients	71 vs. 72	50 vs. 58	386	Mortality	NA	Turkey
Bi (2020)	Hospitalized patients	54 vs. 44	59 vs. 56	113	Severity	9	China
Cekic (2021)	Medical and ICU patients	60 vs. 71	55 vs. 65	590	Mortality	13	Turkey
Gemcioglu (2021)	Hospitalized patients	68 vs. 42	55 vs. 53	301	Severity	10.2	Turkey
Gozdas (2022)	ICU patients	76 vs. 65	60 vs. 57	348	Mortality	NA	Turkey
Kucukceran (2021)	Patient admitted to ED	61 vs. 76	62 vs. 51	717	Mortality	11	Turkey
Kuluozturk (2021)	Hospitalized patients	65 vs. 54	64 vs. 58	400	Mortality	14	Turkey
Mihic (2022)	ICU patients	69 vs. 66	NA	137	Mortality	NA	Croatia
Torun (2021)	Hospitalized patients	62 vs. 60	54 vs. 47	188	Severity	11.4	Turkey
Yang (2021)	Hospitalized patients	61 vs. 53	54 vs. 44	495	Severity	12	China

ED: emergency department; ICU: intensive care unit; NA: not available; F/A ratio: fibrinogen-to-albumin ratio.

## Data Availability

Not applicable.

## Supplementary Materials

The following supporting information can be downloaded at: https://www.mdpi.com/article/10.3390/diagnostics12071678/s1, Table S1: Search strategies for Medline.

## References

[1] Chang D., Chang X., He Y., Tan K.J.K. (2022). The determinants of COVID-19 morbidity and mortality across countries. Sci. Rep..

[2] Perico L., Benigni A., Casiraghi F., Ng L.F.P., Renia L., Remuzzi G. (2021). Immunity, endothelial injury and complement-induced coagulopathy in COVID-19. Nat. Rev. Nephrol..

[3] Guan W.J., Ni Z.Y., Hu Y., Liang W.H., Ou C.Q., He J.X., Liu L., Shan H., Lei C.L., Hui D.S. (2020). Clinical Characteristics of Coronavirus Disease 2019 in China. N. Engl. J. Med..

[4] Huang C., Wang Y., Li X., Ren L., Zhao J., Hu Y., Cao B. (2020). Clinical features of patients infected with 2019 novel coronavirus in Wuhan, China. Lancet.

[5] Reddy S.G.K., Mantena M., Garlapati S.K.P., Manohar B.P., Singh H., Bajwa K.S., Tiwari H. (2021). COVID-2019-2020-2021: Systematic Review and Meta-Analysis. J. Pharm. Bioallied. Sci..

[6] Laino M.E., Ammirabile A., Lofino L., Lundon D.J., Chiti A., Francone M., Savevski V. (2022). Prognostic findings for ICU admission in patients with COVID-19 pneumonia: Baseline and follow-up chest CT and the added value of artificial intelligence. Emerg. Radiol..

[7] Hu H., Pan H., Li R., He K., Zhang H., Liu L. (2022). Increased Circulating Cytokines Have a Role in COVID-19 Severity and Death With a More Pronounced Effect in Males: A Systematic Review and Meta-Analysis. Front. Pharmacol..

[8] Zhu N., Zhang D., Wang W., Li X., Yang B., Song J., Tan W. (2020). A Novel Coronavirus from Patients with Pneumonia in China, 2019. N. Engl. J. Med..

[9] Hajiasgharzadeh K., Jafarlou M., Mansoori B., Dastmalchi N., Baradaran B., Khabbazi A. (2022). Inflammatory reflex disruption in COVID-19. Clin. Exp. Neuroimmunol..

[10] Wang D., Hu B., Hu C., Zhu F., Liu X., Zhang J., Peng Z. (2020). Clinical Characteristics of 138 Hospitalized Patients With 2019 Novel Coronavirus-Infected Pneumonia in Wuhan, China. JAMA.

[11] Xu X.W., Wu X.X., Jiang X.G., Xu K.J., Ying L.J., Ma C.L., Li L.J. (2020). Clinical findings in a group of patients infected with the 2019 novel coronavirus (SARS-Cov-2) outside of Wuhan, China: Retrospective case series. Bmj.

[12] Bi X., Su Z., Yan H., Du J., Wang J., Chen L., Li J. (2020). Prediction of severe illness due to COVID-19 based on an analysis of initial Fibrinogen to Albumin Ratio and Platelet count. Platelets.

[13] Lippi G., Plebani M., Henry B.M. (2020). Thrombocytopenia is associated with severe coronavirus disease 2019 (COVID-19) infections: A meta-analysis. Clin. Chim. Acta.

[14] Göbel K., Eichler S., Wiendl H., Chavakis T., Kleinschnitz C., Meuth S.G. (2018). The coagulation factors fibrinogen, thrombin, and factor XII in inflammatory disorders—A systematic review. Front. Immunol..

[15] Brunclikova M., Simurda T., Zolkova J., Sterankova M., Skornova I., Dobrotova M., Kubisz P. (2022). Heterogeneity of Genotype-Phenotype in Congenital Hypofibrinogenemia—A Review of Case Reports Associated with Bleeding and Thrombosis. J. Clin. Med..

[16] Li Q., Cao Y., Chen L., Wu D., Yu J., Wang H., Hu Y. (2020). Hematological features of persons with COVID-19. Leukemia.

[17] Wool G.D., Miller J.L. (2021). The Impact of COVID-19 Disease on Platelets and Coagulation. Pathobiology.

[18] Hariyanto T.I., Japar K.V., Kwenandar F., Damay V., Siregar J.I., Lugito N.P.H., Kurniawan A. (2021). Inflammatory and hematologic markers as predictors of severe outcomes in COVID-19 infection: A systematic review and meta-analysis. Am. J. Emerg. Med..

[19] Eckart A., Struja T., Kutz A., Baumgartner A., Baumgartner T., Zurfluh S., Schuetz P. (2020). Relationship of Nutritional Status, Inflammation, and Serum Albumin Levels During Acute Illness: A Prospective Study. Am. J. Med..

[20] Xiao L., Jia Y., Wang X., Huang H. (2019). The impact of preoperative fibrinogen-albumin ratio on mortality in patients with acute ST-segment elevation myocardial infarction undergoing primary percutaneous coronary intervention. Clin. Chim. Acta.

[21] Tan Z., Zhang M., Han Q., Wen J., Luo K., Lin P., Fu J. (2017). A novel blood tool of cancer prognosis in esophageal squamous cell carcinoma: The Fibrinogen/Albumin Ratio. J. Cancer.

[22] Mehta P., McAuley D.F., Brown M., Sanchez E., Tattersall R.S., Manson J.J. (2020). COVID-19: Consider cytokine storm syndromes and immunosuppression. Lancet.

[23] Vong T., Yanek L.R., Wang L., Yu H., Fan C., Zhou E., Mullin G.E. (2022). Malnutrition Increases Hospital Length of Stay and Mortality among Adult Inpatients with COVID-19. Nutrients.

[24] Afşin A., Tibilli H., Hoşoğlu Y., Asoğlu R., Süsenbük A., Markirt S., Tuna V.D. (2021). Fibrinogen-to-albumin ratio predicts mortality in COVID-19 patients admitted to the intensive care unit. Adv. Respir. Med..

[25] Çekiç D., Emir Arman M., Cihad Genc A., İşsever K., Yıldırım İ., Bilal Genc A., Yaylacı S. (2021). Predictive role of FAR ratio in COVID-19 patients. Int. J. Clin. Pract..

[26] Gómez C.A., Sun C.K., Tsai I.T., Chang Y.P., Lin M.C., Hung I.Y., Hung K.C. (2021). Mortality and risk factors associated with pulmonary embolism in coronavirus disease 2019 patients: A systematic review and meta-analysis. Sci. Rep..

[27] Hung K.C., Ho C.N., Chen I.W., Hung I.Y., Lin M.C., Lin C.M., Sun C.K. (2021). The impact of aminophylline on incidence and severity of post-dural puncture headache: A meta-analysis of randomised controlled trials. Anaesth. Crit. Care Pain Med..

[28] Hayden J.A., van der Windt D.A., Cartwright J.L., Côté P., Bombardier C. (2013). Assessing bias in studies of prognostic factors. Ann. Intern. Med..

[29] Hung K.C., Wang L.K., Lin Y.T., Yu C.H., Chang C.Y., Sun C.K., Chen J.Y. (2022). Association of preoperative vitamin D deficiency with the risk of postoperative delirium and cognitive dysfunction: A meta-analysis. J. Clin. Anesth..

[30] Higgins J.P., Thompson S.G., Deeks J.J., Altman D.G. (2003). Measuring inconsistency in meta-analyses. Bmj.

[31] Takwoingi Y., Riley R.D., Deeks J.J. (2015). Meta-analysis of diagnostic accuracy studies in mental health. Evid. Based Ment. Health.

[32] Gemcioglu E., Davutoglu M., Catalbas R., Karabuga B., Kaptan E., Aypak A., Ates I. (2021). Predictive values of biochemical markers as early indicators for severe COVID-19 cases in admission. Future Virol..

[33] Gozdas H.T., Kayis S.A., Damarsoy T., Ozsari E., Turkoglu M., Yildiz I., Demirhan A. (2022). Multi-inflammatory Index as a Novel Mortality Predictor in Critically Ill COVID-19 Patients. J. Intensive Care Med..

[34] Küçükceran K., Ayranci M.K., Girişgin A.S., Koçak S. (2021). Predictive value of D-dimer/albumin ratio and fibrinogen/albumin ratio for in-hospital mortality in patients with COVID-19. Int. J. Clin. Pract..

[35] Kuluöztürk M., Deveci F., Turgut T., Öner Ö. (2021). The Glasgow Prognostic Score and fibrinogen to albumin ratio as prognostic factors in hospitalized patients with COVID-19. Expert Rev. Respir. Med..

[36] Mihić D., Maričić L., Tolj I., Loinjak D., Sušić L., Begić I. (2022). Prognostic significance inflammatory scoring systems in critically ill patients with COVID-19 infection. Med. Jadertina.

[37] Torun A., Çakırca T.D., Çakırca G., Portakal R.D. (2021). The value of C-reactive protein/albumin, fibrinogen/albumin, and neutrophil/lymphocyte ratios in predicting the severity of COVID-19. Revista da Associação Médica Brasileira.

[38] Yang R., Gui X., Ke H., Gao S., Luo M., Xiong Y. (2021). The indicative role of markers for liver injury on the severity and prognosis of coronavirus disease 2019 patients. Eur. J. Gastroenterol. Hepatol..

[39] McGovern J., Al-Azzawi Y., Kemp O., Moffitt P., Richards C., Dolan R.D., Maguire D. (2022). The relationship between frailty, nutritional status, co-morbidity, CT-body composition and systemic inflammation in patients with COVID-19. J. Transl. Med..

[40] Cvachovec K., Horácek M., Vislocký I. (2000). A retrospective survey of fibrinolysis as an indicator of poor outcome after cardiopulmonary bypass and a possible early sign of systemic inflammation syndrome. Eur. J. Anaesthesiol..

[41] Tousoulis D., Papageorgiou N., Androulakis E., Briasoulis A., Antoniades C., Stefanadis C. (2011). Fibrinogen and cardiovascular disease: Genetics and biomarkers. Blood Rev..

[42] Karahan O., Yavuz C., Kankilic N., Demirtas S., Tezcan O., Caliskan A., Mavitas B. (2016). Simple blood tests as predictive markers of disease severity and clinical condition in patients with venous insufficiency. Blood Coagul. Fibrinolysis.

[43] Simurda T., Asselta R., Zolkova J., Brunclikova M., Dobrotova M., Kolkova Z., Kubisz P. (2021). Congenital Afibrinogenemia and Hypofibrinogenemia: Laboratory and Genetic Testing in Rare Bleeding Disorders with Life-Threatening Clinical Manifestations and Challenging Management. Diagnostics.

[44] Simurda T., Vilar R., Zolkova J., Ceznerova E., Kolkova Z., Loderer D., Kubisz P. (2020). A Novel Nonsense Mutation in FGB (c.1421G>A; p.Trp474Ter) in the Beta Chain of Fibrinogen Causing Hypofibrinogenemia with Bleeding Phenotype. Biomedicines.

[45] Nugroho J., Wardhana A., Mulia E.P., Maghfirah I., Rachmi D.A., A’Yun M.Q., Septianda I. (2021). Elevated fibrinogen and fibrin degradation product are associated with poor outcome in COVID-19 patients: A meta-analysis. Clin. Hemorheol. Microcirc..

[46] Cabrerizo S., Cuadras D., Gomez-Busto F., Artaza-Artabe I., Marín-Ciancas F., Malafarina V. (2015). Serum albumin and health in older people: Review and meta analysis. Maturitas.

[47] Chen C., Wang S., Chen J., Liu X., Zhang M., Wang X., Si M. (2019). Escin suppresses HMGB1-induced overexpression of aquaporin-1 and increased permeability in endothelial cells. FEBS Open Bio..

[48] Cao B., Jing X., Liu Y., Wen R., Wang C. (2022). Comparison of laboratory parameters in mild vs. severe cases and died vs. survived patients with COVID-19: Systematic review and meta-analysis. J. Thorac. Dis..

[49] Stavileci B., Ereren E., Özdemir E., Özdemir B., Cengiz M., Enar R. (2022). The impact of daily troponin I and D-dimer serum levels on mortality in COVID-19 pneumonia patients. Cardiovasc. J. Afr..

[50] Fathalla L.A., Kamal L.M., Salaheldin O., Khalil M.A., Kamel M.M., Fahim H.H., El-Meligui Y.M. (2022). Laboratory biomarker predictors for disease progression and outcome among Egyptian COVID-19 patients. Int. J. Immunopathol. Pharmacol..

[51] Sahu B.R., Kampa R.K., Padhi A., Panda A.K. (2020). C-reactive protein: A promising biomarker for poor prognosis in COVID-19 infection. Clin. Chim. Acta.

[52] Chen Z., Zhang F., Hu W., Chen Q., Li C., Wu L., Yue J. (2021). Laboratory markers associated with COVID-19 progression in patients with or without comorbidity: A retrospective study. J. Clin. Lab. Anal..

[53] Tirupathi R., Muradova V., Shekhar R., Salim S.A., Al-Tawfiq J.A., Palabindala V. (2020). COVID-19 disparity among racial and ethnic minorities in the US: A cross sectional analysis. Travel Med. Infect. Dis..

[54] Mackey K., Ayers C.K., Kondo K.K., Saha S., Advani S.M., Young S., Kansagara D. (2021). Racial and Ethnic Disparities in COVID-19-Related Infections, Hospitalizations, and Deaths: A Systematic Review. Ann. Intern. Med..

[55] Liu X., Zhou H., Zhou Y., Wu X., Zhao Y., Lu Y., Wang Y. (2020). Risk factors associated with disease severity and length of hospital stay in COVID-19 patients. J. Infect..

[56] Qian Z., Li Z., Peng J., Gao Q., Cai S., Xu X. (2022). Association between hypertension and prognosis of patients with COVID-19: A systematic review and meta-analysis. Clin. Exp. Hypertens..

[57] Kastora S., Patel M., Carter B., Delibegovic M., Myint P.K. (2022). Impact of diabetes on COVID-19 mortality and hospital outcomes from a global perspective: An umbrella systematic review and meta-analysis. Endocrinol. Diabetes Metab..

