# Supplementary material for: Association between Fibrinogen-to-Albumin Ratio and Prognosis of Hospitalized Patients with COVID-19: A Systematic Review and Meta-Analysis
Source: Diagnostics (Basel). 2022 Jul 10;12(7):1678. doi: 10.3390/diagnostics12071678 (PMC9317445; doi:10.3390/diagnostics12071678)
Supplement: Supplementary file 1 [file diagnostics-12-01678-s001.zip › diagnostics-1808247-supplementary.pdf]

Table S1. Search strategies for Medline.

1	("severe acute respiratory syndrome" or "coronavirus 2" or "coronavirus" or "corona virus" or "covid-19" or "nCoV" or "2019nCoV" or "Wuhan virus" or "2019-nCoV" or "SARS-CoV-2 Infection*").mp.
2	exp "COVID 19"/ or exp "SARS-CoV-2"/
3	("fibrinogen-to-albumin ratio" or "fibrinogen to albumin ratio").mp.
4	(1 or 2) and 3
